# Editorial: Novel Nanotechnology for Diagnosing and Treating Eye Disorders

**DOI:** 10.3389/fbioe.2021.639230

**Published:** 2021-02-10

**Authors:** Yuqing Wu, Lei Gu, Yujia Cai, Yiyun Cheng, Jiaxu Hong

**Affiliations:** ^1^Department of Ophthalmology and Vision Science, Shanghai Eye and ENT Hospital, Fudan University, Shanghai, China; ^2^Max Planck Institute for Heart and Lung Research and Cardiopulmonary Institute (CPI), Bad Nauheim, Germany; ^3^Shanghai Center for Systems Biomedicine, Shanghai Jiao Tong University, Shanghai, China; ^4^Shanghai Key Laboratory of Regulatory Biology, School of Life Sciences, East China Normal University, Shanghai, China

**Keywords:** nanotecehnology, diagnosis, treatment, eye disorders, biomaterial

In recent years, novel nanotechnology has advanced dramatically in ophthalmology, and has contributed to our understanding of eye physiology, diagnosis of eye disorders, and treatments of eye diseases. The Research Topic “Novel Nanotechnology for Diagnosing and Treating Eye Disorders” includes one perspective and three original researches with the purpose of maximizing our understanding of nanomedicine in drug delivery and materials for tissue engineering.

Great advances have been made in nanoparticles regarding drug delivery systems in the ophthalmic field. In this perspective article (Yu et al.), describe the polysaccharide-based nanomaterials in detail. Firstly, the authors discuss the structural instability of polysaccharide-based nanocarriers, which needs to be overcome during production, especially for polysaccharides with branched-chain or special functional groups. Secondly, they point out the potential for developing the bioactive polysaccharide-based ocular drug nanocarriers that can serve as carrier and play therapeutic functions. Thirdly, they review four typical ocular drug delivery nanomaterials based on the types of geometric structures, from zero- to three-dimensional. They also propose strategies to maximize drug delivery capacity, such as using hybrid nanomaterials, developing polysaccharide-based cell-loading nanocarriers, introducing novel structure types, and synthesizing nanoparticles based on green chemistry. Lastly, they summarize the behavior of nanomaterials in ocular tissues.

Previous research has demonstrated the usefulness of novel ophthalmic formulations containing tranilast nanoparticles (ophthalmic TL-NPs formulations), which showed high drug adhesion to ocular tissues. Here (Nagai et al.), designed an *in situ* gel incorporating TL-NPs with 0.5–3% methylcellulose (MC, type SM-4) to ensure long residence time and sustained release of the drug on the ocular surface. In terms of drug behavior, an *in vivo* study showed that the gel formulations of TL-NPs with 0.5 and 1.5% MC prolonged drug residence time and increased TL uptake into cornea and conjunctiva when compared to TL-NPs with or without 3% MC. As to therapeutic efficacy, a stronger anti-inflammatory effect was observed in TL-NPs with the optimum amount of MC (0.5–1.5%) rather than with excessive MC (3%). These findings could give insights into the design of studies in the field of ophthalmic nanomedicines.

Keratomycosis is a severe eye disease, usually caused by fungal infection. Current antimycotic drugs such as Amphotericin B (AmpB) are still hard to formulate for fungal keratitis treatment, due to their low solubility and stability in aqueous media. Based on previous studies Göttel et al., developed the *in situ* gelling consisting of pullulan and gellan gum with AmpB. In this research, the drug was spun without any further solubilization ingredient (IIa), after addition of sodium cholate (IIb), drug-loaded PLGA nanoparticles (IIc), and using a polyelectrolyte complex (IId). Among them, the complex-loaded fibers (IId) showed a desirable inhibition of fungal growth, while IIb failed to inhibit fungal strain *Issatchenkia orientalis* effectively. In addition to its anti-fungal activity, Amphotericin B polyelectrolyte complex (AmpB-Eu L) displayed good cell tolerance during *in vivo* experiments and sufficient resistance against degeneration induced by irradiation-sterilization. Hence, AmpB-Eu L could be a new and promising option for fungal keratitis therapy.

Nanotechnology has also been addressed in tissue engineering. Amniotic membrane (AM) is abundant in the source of growth factors (GF), and AM transplantation is used to promote epithelialization and wound healing in surgical treatment. However, traditional AM is far from satisfactory effects due to its short shelf life Here (Zhao et al.), reported that modified AM (AM-HEP@EGF), with surface grafting heparin combined with epithelial growth factor (EGF), has the advantage of long storage time and sustained release of GF. On the one hand, experiments *in vitro* have demonstrated the biocompatibility and ability of the modified AM to promote corneal epithelial cell growth and migration. On the other hand, the results from *in vivo* experiments showed that compared to other groups, AM-HEP@EGF had better anti-inflammatory effects, and achieved higher transparency as well as epithelialization in the treated cornea. Therefore, AM-HEP@EGF could be a feasible alternative in corneal alkali burns treatment or accelerating epithelial wound healing.

In this topic, we discussed nanomedicine and biomaterials in terms of drug delivery systems and materials for tissue engineering, such as *in situ* gel formulations with nanoparticles and modified AM. In conclusion, therapy originating from nanomedicine has contributed largely to enhanced effects. For better understanding of this promising field, more studies are required in the future.

## Author Contributions

All authors listed have made a substantial, direct and intellectual contribution to the work, and approved it for publication.

## Conflict of Interest

The authors declare that the research was conducted in the absence of any commercial or financial relationships that could be construed as a potential conflict of interest.

